# Recommendations for long-term follow-up care of secondary health conditions in spinal cord injury/disorder: a systematic review

**DOI:** 10.3389/fresc.2024.1371553

**Published:** 2024-10-11

**Authors:** Inge Eriks-Hoogland, Xavier Jordan, Michael Baumberger, Vanessa Seijas, Burkhart Huber, Franz Michel, Roland Thietje, Lorena Müller

**Affiliations:** ^1^ Department of Paraplegiology, Swiss Paraplegic Centre (SPZ), Nottwil, Switzerland; ^2^ Faculty of Health Sciences and Medicine, University of Lucerne, Lucerne, Switzerland; ^3^ Department of Health Services and Clinical Care, Swiss Paraplegic Research (SPF), Nottwil, Switzerland; ^4^ Department of Paraplegiology, Clinique Romande de Réadaptation, Sion, Switzerland; ^5^ Department of Traumatology, AUVA Rehabilitation Centre, Häring, Austria; ^6^ Department of Paraplegiology, REHAB Basel, Basel, Switzerland; ^7^ Department Neuro-Urology, Centre for Spinal Injuries, BG Trauma Hospital Hamburg, Hamburg, Germany

**Keywords:** spinal cord injury, spinal cord disease, spina bifida, follow-up care, lifelong follow-up, outpatient care, morbidity

## Abstract

**Objectives:**

The purpose of this systematic review is to provide an overview of published follow-up care programs of primary and secondary health conditions (SHCs) in spinal cord injury/disorder (SCI/D) and spina bifida and describe recommendations on content, frequency, setting of follow-up care programs for persons with SCI/D and spina bifida.

**Methods:**

According to the sequence of procedures of the AWMF (Association of the Scientific Medical Societies in Germany) a systematic literature search was performed (in PubMed, Cochrane Library and nine additional databases for guidelines) between 5 September 2019 and 22 September 2019. Publications (Jan. 2008–Dec. 2018) and guidelines (up to 2018) published in English or German and describing an evidence-based follow-up care program for persons with SCI/D or spina bifida were included.

**Results:**

The systematic literature search found 1973 publications in PubMed and Cochrane Library, resulting in 19 papers for SCI/D and 6 for Spina bifida. Additionally, we included 34 guidelines developed by reputable committees or medical associations. All eligible guidelines, and publications, were rated and classified according to the guidance of AWMF. Of the retrieved publications, and guidelines, level of evidence of follow-up care programs was mostly based on informal procedures and expert opinion or formally consent based expert opinion. None of the guidelines, or publications described an evidence based comprehensive clinical practice guideline (CPG) for follow-up care for people with SCI/D or spina bifida.

**Conclusion:**

Based on the comprehensive and extensive literature research conducted, regular (annual) follow-up care appointments at specialized SCI clinics are recommended. There is a notable absence of a comprehensive CPG covering all relevant health conditions for long-term follow-up in SCI/D or spina bifida. In order to provide persons with SCI/D with up-to-date and best possible medical and rehabilitative care, a CPG for follow-up care is urgently needed. In response to this gap, the German-speaking Medical Society of Paraplegia (DMGP) has commissioned its members to establish a guideline for follow-up care for individuals with SCI/D. The current review serves as an evidence-based framework for the development of this guideline.

## 1 Introduction

A spinal cord injury/disorder (SCI/D) represents a chronic and complex medical condition. This condition can result from trauma, disease (e.g., spina bifida, tumor, vascular disease), or degenerative disorders. Along the continuum of care, persons with SCI/D are challenged with many consequences such as loss of muscle power, sensory function, and an increased risk of secondary health conditions (SHCs) (1–3). These SHCs, which encompass a spectrum of physical and physiological complications, are described to be related to higher age and cause of SCI (more often in NTSCI) (4), but some (such as respiratory problems and pressure injuries) are also related to level and completeness of SCI. SHCs can significantly impact overall well-being and quality of life. Among the most prevalent SHCs observed in persons with SCI are urinary tract infections, pressure injuries, and respiratory infections (4–6). Urinary tract infections can be particularly troublesome due to the disruption of normal bladder function and impaired immune responses, making individuals with SCI/D more susceptible to these infections. Pressure injuries result from prolonged immobility and the loss of sensation in affected areas, leading to tissue breakdown and the formation of open wounds. Respiratory infections often arise due to weakened respiratory muscles and compromised cough reflexes, making individuals with SCI/D vulnerable to pneumonia and other respiratory ailments.

A comprehensive survey conducted within the Swiss SCI/D population unveiled the presence of an average of seven concurrent health conditions per individual, and prevalence of health conditions increased with age and was higher in non-traumatic SCH (4). This presence of multimorbidity in the SCI population not only exacerbates functional impairment but also imposes a considerable burden on healthcare systems, resulting in elevated healthcare expenditures. Consequently, individuals with SCI/D require continuous medical care, leading to a heightened rate of healthcare service utilization when compared to the general population (7).

Follow-up care programs in specialized SCI clinics target to prevent or early diagnose those SHCs. These programs emphasize the importance of regular check-up appointments for effective healthcare management (8, 9). The rationale behind these check-ups lies in their potential to detect SHCs at an early, more manageable stage, ultimately enhancing the long-term health, preventing costly inpatient treatments and enhancing functioning and well-being of persons with SCI/D (10).

Regarding above, findings from a Swiss community survey conducted in 2017/18 have revealed a concerning statistic: only 51% of all individuals with SCI adhere to annual follow-up appointments (11). Furthermore, research has demonstrated that the consequences of not adhering to follow-up care in specialized SCI clinics can be severe. In a study by Chamberlain et al. (12) it was found that persons with traumatic SCI who had not received treatment at a specialized center for follow-up care faced a substantially heightened risk of mortality (hazard ratio: 3.62 with CI 2.18–6.02). This statistic emphasizes the critical role that specialized SCI clinics play in not only managing SHCs but also in preserving the overall health, functioning, and life expectancy of individuals with SCI.

However, despite the clear benefits of regular check-ups up to now, limited evidence regarding content and frequency of follow-up care programs for persons with SCI/D exist (13). In 2005 Bloemen-Vrencken, de Witte & Post performed a literature study regarding follow-up care in SCI and described the effects of different follow-up care programs regarding secondary impairments, well-being of individuals, quality of care provided, and the associated costs. This review disclosed that, in 2005, hardly any evidence existed regarding content and frequency of follow-up care in persons with SCI/D. If described, the content of these descriptions was often focusing on one secondary health condition, based on expert opinion, and not specific. The researchers pointed out the urgent need for the development and publication of comprehensive follow-up care programs tailored specifically for individuals with SCI/D.

The current systematic review was undertaken to explore and describe current evidence on long-term follow-up care of SHCs in SCI/D or spina bifida and to serve as a basis for the development of a clinical practice guideline (CPG) for follow-up care as commissioned by the German-speaking medical SCI Society (DMGP). Although we are aware that follow-up care in SCI and spina bifida is much broader than follow-up of HCs, the current review was limited to this topic. The objective of this systematic review therefore is to provide an overview of current evidence and recommendation regarding follow-up care for SHCs in SCI/D and spina bifida with following specific aim: To describe current existing recommendations on content, frequency, setting of follow-up care programs focusing on SHCs for persons with SCI/D and spina bifida.

## 2 Methods

### 2.1 Study design, information sources and search strategy

We conducted a systematic review of clinical practice guidelines (CPGs) and publications using the JBI Manual for Evidence Synthesis (14). The search was conducted in all databases between 5 September 2019 and 22 September 2019 and included publications from January 2008 until December 2018.

Publications and guidelines were selected from PubMed database and Cochrane Library. For our search we used following search terms: (1a) spinal cord injuries/disorder (ie paraplegia, tetraplegia) or (1b) spina bifida, (2) secondary health problems (ie secondary impairments, medical problems), and (3) follow-up care (ie long-term-care, outpatient care). The detailed search strategy is described in Supplementary Material (Table 1). We filtered for results in Human Studies, written in English and German, and published between 2008 and 2018. In the literature, aspects for spina bifida are often covered under aspects for non-traumatic spinal cord injury (15, 16). This leads to underrepresentation of relevant aspects for this population. Therefore, we added a separate search for spina bifida in our search strategy to ensure that aspects, relevant for persons with spina bifida are also covered in the review.

**Table 1:** Inclusion criteria according to PICO format.

Criteria	Description
Population	Persons with SCI/SCD or spina bifida in follow-up care
Interventions	Examination/outcome assessment for neurological, general medical/internal medicine, urogenital and musculoskeletal aspects
Comparators	No comparator
Outcomes	Recommendations concerning the content and frequency of follow-up care
Study design	All

SCI, spinal cord injury; SCD, spinal cord disorder.

In addition, based on expert opinion and a google search, we searched following databases, or websites for guidelines on the topic of follow-up care, published up to 2018: (1) Association of the Scientific Medical Societies in Germany (AWMF), (2) Spinal Cord Injury Research Evidence (SCIRE), (3) The National Institute for Health and Care Excellence (NICE), (4) National Guideline Clearinghouse practice guidelines (website no longer available), (5) British Society of Rehabilitation medicine, (6) Clinical practice Guideline for persons with SCI, (7) Cochrane, (8) Guidelines international network, (9) American Spina Bifida Association, (10) Guidelines in PubMed. The search strategy for those guidelines is provided in Supplementary Material (Table 2).

**Table 2:** Medical content of guidelines and publications.

Database	Title	Medical issues/content													Patient	
		Bowel (b510, b540, b810, b820, s540)	Bladder (b610-b639)	Pain (b280)	Pressure Injury (s810, b810, b820)	Osteoporosis (b729)	Pregnancy (b640, b660)	Sexuality (b640)	Cardiometabolic System (b410-b429, b540)	Lower and upper limp (b7, s760)	Respiratory System (b440-b449)	Psychological Issues (b130)	Thromboembolism (b430)	Rehabilitation	Spinal cord injury	Spina bifida
AWMF	Schwangerschaft, Geburt und Wochenbett bei Frauen mit Querschnittlähmung (S2k) (17)						**x**								**x**	
	Schmerzen bei Querschnittlähmung (S2k) (18)			**x**											**x**	
	Querschnittspezifische Dekubitusbehandlung und –prävention (S1) (19)				**x**										**x**	
	Rehabilitation der unteren Extremität, der Steh- und Gehfunktion bei Menschen mit Querschnittlähmung (S2e) (20)									**x**					**x**	
	Querschnittlähmungsassoziierte Osteoporose (S1) (21)					**x**									**x**	
	Neurogene Darmfunktionsstörung bei Querschnittlähmung (S2k) (22)	**x**													**x**	
	Depression bei Menschen mit Querschnittlähmung: Besonderheiten in der Diagnostik und Behandlung (S1) (23)											**x**			**x**	
	Diagnostik und Therapie der neurogenen Blasenfunktionsstörungen bei Kindern und Jugendlichen mit spinaler Dysraphie (S2k) (24)		**x**													**x**
	Neuro-urologische Versorgung querschnittgelähmter Patienten (S2k) (25)		**x**												**x**	
SCIRE	Rehabilitation Practices (26)													**x**	**x**	**x**
NICE	Urinary incontinence in neurological disease: assessment and management (27)		**x**												**x**	**x**
	Faecal incontinence in adults: management (28)	**x**													**x**	**x**
	Pressure ulcers: prevention and management (29)				**x**										**x**	
Guidelines British Society of Reha-bilitation Medicine	BSRM Standards for Rehabilitation Services mapped on to the NSF for Long-term neurological conditions (30)													**x**	**x**	
Clinical practice guidelines for persons with spinal cord injury	Prevention of Venous Thromboembolism in Individuals with Spinal Cord Injury (31)												**x**		**x**	
	Sexuality and Reproductive Health in Adults with Spinal Cord injury (32)							**x**							**x**	
	Bladder Management for Adults with Spinal Cord Injury (33)		**x**												**x**	
	Preservation of Upper Limb Function Following Spinal Cord Injury (34)									**x**					**x**	
	Respiratory Management Following Spinal Cord Injury (35)										**x**				**x**	
	Pressure Ulcer Prevention and Treatment Following Spinal Cord Injury, 2nd edition (36)				**x**										**x**	
	Outcomes Following Traumatic Spinal Cord Injury (37)													**x**	**x**	
	Depression Following Spinal Cord Injury (38)											**x**			**x**	
	Neurogenic Bowel Management in Adults with Spinal Cord Injury (39)	**x**													**x**	
	Identification and Management of Cardiometabolic Risk after Spinal Cord Injury (40)								**x**						**x**	
Cochrane	Management of faecal incontinence and constipation in adults with central neurological diseases (41)	**x**													**x**	
	Organisation of health services for preventing and treating pressure ulcers (42)				**x**										**x**	
	Automated telephone communication systems for preventive healthcare and management of long-term conditions (43)														**x**	
American Spina Bifida Association	Guidelines for the Care of People with Spina Bifida (4th edition) (44)	**x**	**x**	**x**	**x**	**x**	**x**	**x**	**x**	**x**	**x**	**x**	**x**			**x**
PubMed	Management of pain in individuals with spinal cord injury: Guideline of the German-Speaking Medical Society for Spinal Cord Injury (45)			**x**											**x**	
	Identification and Management of Cardiometabolic Risk after Spinal Cord Injury: Clinical Practice Guideline for Health Care Providers. (40)								**x**						**x**	
	Urodynamics in patients with spinal cord injury: A clinical review and best practice paper by a working group of The International Continence Society Urodynamics (46)		**x**												**x**	
	Neurogenic bowel dysfunction: Clinical management recommendations of the Neurologic Incontinence Committee of the Fifth International Consultation on Incontinence (47)	**x**													**x**	
	The CanPain SCI Clinical Practice Guidelines for Rehabilitation Management of Neuropathic Pain after Spinal Cord: screening and diagnosis recommendations (48)			**x**											**x**	
	Professional standards of practice for psychologists, social workers, and counselors in SCI rehabilitation (49)											**x**		**x**	**x**	
	Physical and rehabilitation medicine (PRM) care pathways: “spinal cord injury”. (50)													**x**	**x**	
	Spina Bifida Health-care Guidelines for Men's Health (51)							**x**								**x**
	Duplicates			**1**					**1**						**1**	
	Total: 36	**6**	**6**	**3**	**5**	**2**	**2**	**3**	**2**	**3**	**2**	**4**	**2**	**5**	**31**	**6**
	Total SCI/D and spina bifida														**34**	

Bold values in the table represent the frequency of guidelines and publications addressing specific health issue, categorized according to the ICF framework.

### 2.2 Selection procedure and eligibility criteria

Eligibility criteria for publications and guidelines were defined according to the extended PICO-(patient, intervention, comparison, outcome, study design) format (Table 1). We included an article if it described recommendations for medical follow-up care for persons with SCI/D or spina bifida (living in the community). Regarding the study type, we considered guidelines, reviews, interventional studies, observational studies, and opinion papers. If an article focused solely on recommendations for acute phase, nursing, or therapeutic aspects they were excluded. Additionally, we excluded publications considering mainly pediatric aspects, work and employment, or housing and attendant care.

Two reviewers (IEH, an experienced SCI/D physician & LM, a health scientist) assessed the retrieved records by title and abstract against the inclusion criteria. After an initial selection of the literature, the full articles were read and evaluated independently by the two reviewers (IEH & LM). By analyzing the entire text, they decided whether the study met the established criteria. In case of disagreement, discrepancies were solved through discussion or involving a member (all senior physicians) of the core group of the development of the clinical practice guideline for long term follow-up care *n* SCI as a third reviewer (XJ, MB, RT, HB).

### 2.3 Data extraction

Two trained authors (IEH and LM) participated in the data extraction and methodological assessment process using a standardized data extraction form in MS Excel. We retrieved the following information (1) title, (2) year of publication, (3) author, (4) country, (5) publication type, (6) topic (e.g., pressure injury, urology, telemedicine), (7) setting, (8) objective. For guidelines we extracted additional information about recommendations including assessment recommendations.

### 2.4 Methodological assessment and evaluation of follow-up care programs

Of all included manuscripts we extracted aim, method/design (including population, and if applicable intervention, and outcome measures), and results (including recommendations).

IEH and LM evaluated all included articles with the appropriate quality assessment tool: (1) The Strengthening The Reporting of Observational Studies in Epidemiology (STROBE) statement includes quality criteria for different types of observational studies (52). (2) The R-AMSTAR (Revised—A MeaSurement Tool to Assess Systematic Reviews) tool to assess the methodological quality of systematic reviews (53). (3) The German Instrument for Methodological Guideline Appraisal (DELBI) for Guidelines which is based on AGREE (Appraisal of Guidelines for Research and Evaluation) (54). (4) The Checklist for randomized controlled trials of the Joanna Briggs Institute (JBI) was used to assess whether the randomized controlled trials presented reliable and meaningful results for use in clinical practice (55). Opinion papers and qualitative studies were not specifically rated, but we described their content using the above-mentioned categories.

### 2.5 Synthesis methods

The synthesis includes all medical aspects covered in the papers and guidelines (Supplementary Table S1). As a reference framework the ICF-Core Set for long-term care (56) was used to identify and quantify the underlying medical conditions. Following the standardized ICF linking rules (57), two authors (IE and LM) linked the ICF categories of the ICF-Core Set for long-term care to the inherent health condition.

## 3 Results

### 3.1 Study selection

**Figure 1:**
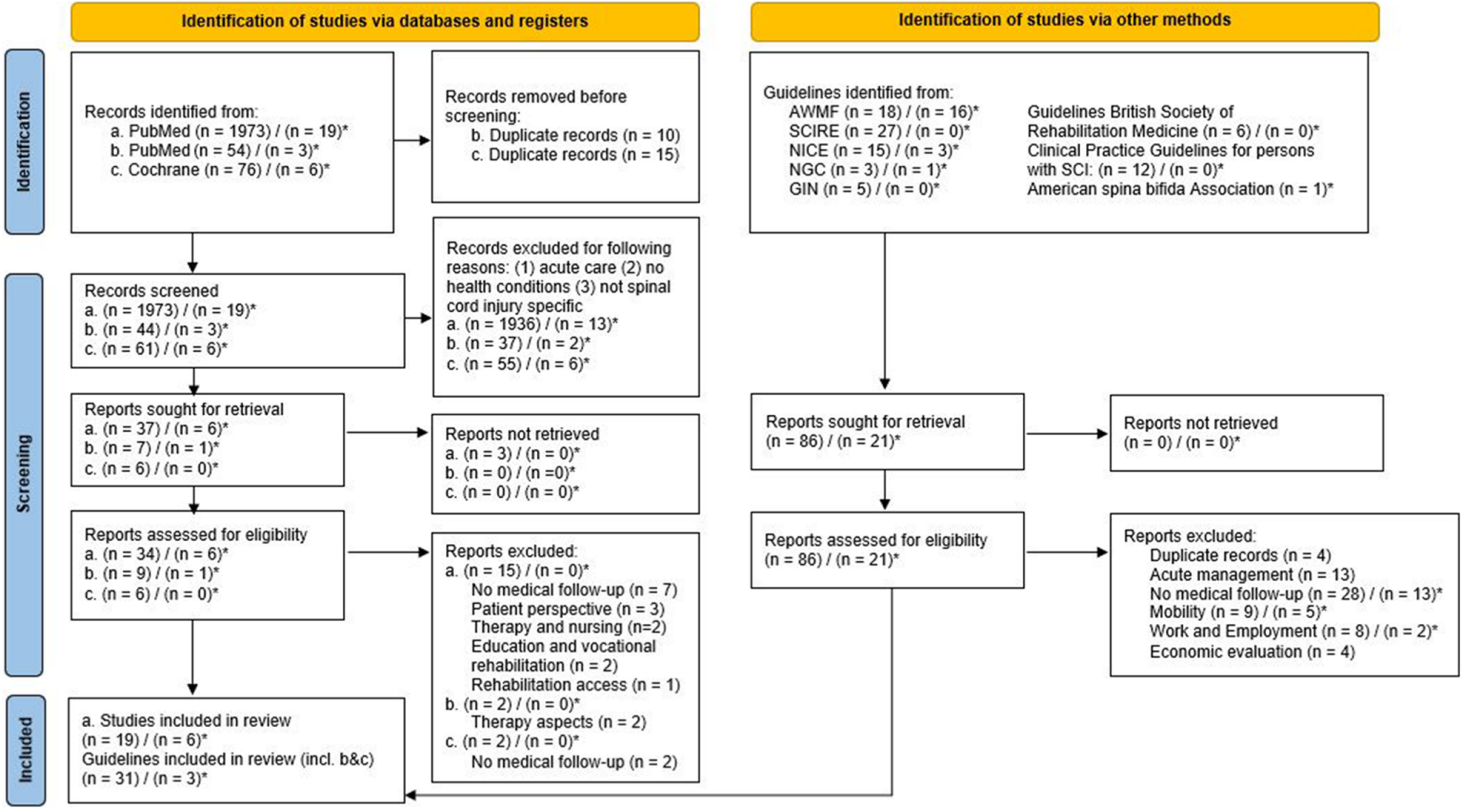
PRISMA flowchart for search strategy and results. **(a)** PubMed search with no specific filters for publication methods. **(b)** PubMed search with limitation on Guidelines. **(c)** Cochrane search. () publications for people with spinal cord injury/disorder. ()* publications specifically for people with spina bifida.

The search for publications related to follow-up care for people with SCI/D was carried out on 5th September 2019 and resulted in 1973 articles and for people with spina bifida in 19 articles. The decision tree for the selection of manuscripts is presented in Figure 1. We finally included 19 articles on SCI/D and 6 articles on spina bifida. The search results are available on request. The search results for guidelines is explained in chapter 3.5.

Most articles were excluded due to missing and no clear conclusion on follow-up care (7), along with some articles focused only on therapeutic and nursing (2) aspects. Others described solely the patient perspective (with focus on quality of life) (3), focusing on consumer needs and highlighted issues of communication. Furthermore, we excluded articles discussing education and vocational rehabilitation (2), as well as one publication focusing on rehabilitation access (1).

### 3.2 Study characteristics

The publications included differ broadly in terms of their content and scope. The articles cover a broad range of medical health conditions, two described urological follow-up (58, 59), one follow-up of osteoporosis (60), and two articles describing follow-up of pressure injuries (61, 62). Additionally, six articles depict aspects and needs in medical services (63–68). These articles provide insights into various aspects of medical services, such as patient-centered care, rehabilitation, and quality improvement. Seven articles discuss follow-up planning (Stiens et al. (9), Morse et al. (60), Spreyermann et al. (67), Saur and Abel (68), McColl et al. (69), Spreyermann and Michel (70) and Van de Pol et al. (71). These articles provide information on different aspects of follow-up planning, such as the development of follow-up protocols, the use of technology in follow-up, and the evaluation of follow-up outcomes. Finally, seven articles evaluate the results of telephone or video-based follow-up or care planning (Van de Pol et al. (71), Hossain et al. (72), Careau et al. (73), Dallolio et al. (74) and Young-Hughes and Simbartl (62). These articles provide insights into the effectiveness of telephone or video-based follow-up or care planning in various medical contexts.

The search results revealed several publications that specifically focus on follow-up care in persons with spina bifida. One review by Dicianno et al. (75) outlines the rehabilitation and medical management of adults with spina bifida. Two publications are based on surveys, one with urologists elucidating current practices regarding urological management on long-term follow-up after childhood (76), and the other conducted with middle-aged persons with spina bifida about medical and psychosocial problems (77). Two articles evaluate aspects of medical services (78, 79), and one describes medical needs, specifically the correlation of childhood health condition related to spina bifida and the specialized consultations in adulthood (80).

### 3.3 Target population and total sample size

The number of persons with SCI/D included in the selected articles varied from 28 to 167'600. Seven articles did not mention the number of participants, as this was not applicable due to the study design, such as opinion papers or guidelines. The target population in all follow-up care programs were persons with SCI/D solely, except in the cohort study from Mitchell et al. (64). In two programs, the perspectives of care providers were also included (66, 67). Notably, four publications (60–62, 81) focused on veterans of America (VA), while one publication collected data from both VA and civilians with SCI (65).

In publications about topics of persons with spina bifida the number of participants varied from 38 to 2016. Described were persons with spina bifida (75, 77, 79), Myelomeningocele (80), but also individuals with general neurological condition (78). All but one publication focused on the patient perspective, while Szymanski et al. (76) summarizes the perspective of pediatric urologists for long-term follow-up of patients with congenital genitourinary conditions.

### 3.4 Setting (methods) and frequency of follow-up care

Regarding the setting for follow-up care, a minority of publications focused on check-up visits in specialized SCI/D centers (9, 82). Two papers emphasized the role of family physicians as the main coordinators in long term health care of persons with SCI/D (63, 69). The majority of the papers recommended conducting a broad range of medical services, such as urology and gynecology, coordinated by a SCI/D specialist (9, 64, 68, 70). Telemedicine as a solution for follow-up care has been described in few papers in various healthcare settings (Canada, Australia, Europe, Bangladesh, and USA). Some papers highlighted the importance of telemedicine (73, 74, 81) as an option to diagnose and treat patients remotely. According to these publications, particularly for people living in remote areas (71) or in low- and middle-income countries (72) telemedicine might provide access to healthcare services. Furthermore, while all publications stress the importance of ongoing lifelong care for persons with SCI/D, they differ in their recommendations about the frequency of check-ups. Some advocate for annual check-ups (67, 69, 82), while others highlight the importance of risk-modified frequency, which may be determined by factors such as injury level and associated diagnosis (9, 25, 59, 63).

Regarding the setting of follow-up care for persons with spina bifida the recommendations focus on life-long and multispecialized follow-up care (75, 77, 80). Veenboer et al. (77) propose that rehabilitation physicians act as a coordinator and gatekeeper for more specialist care. Additionally (76), recommends urologists as the most appropriate health professionals. Bakketun et al. (80) conducted a retrospective cohort study to investigate the healthcare setting in which consultations took place for patients with myelomeningocele (MMC), finding that most consultations occurred in the outpatient setting, with gastroenterology being the most common specialty. However, the highest hospitalization rate was found in medical issues related to neurosurgical problems. Regarding the frequency of follow-up care for patients with MMC only Veenboer et al. (77) provides a recommendation. The authors recommend that regular visits to an outpatient clinic should occur every 18–24 months.

### 3.5 Guidelines selection and characteristics

A total of 34 guidelines were found including the topic of follow-up care for SCI/D (see Figure 1). In total 31 guidelines are focusing on people with SCI/D, three guidelines solely on people with spina bifida, and another three guidelines address both people with spina bifida and SCI/D.

Most guidelines focused on a specific medical issue with the most frequent topics (6 guidelines) being bowel (22, 28, 39, 41, 47) and bladder management (24, 25, 27, 33, 44, 46). Almost as many guidelines could be found on the subject of pressure injuries (5 guidelines) (19, 29, 36, 42, 44) and rehabilitation practices and services in the long term context (26, 30, 37, 44, 49, 50). Other topics covered by different guidelines include pain (18, 45, 48), respiratory system (2 guidelines) (35, 44), cardiometabolic system (2 guidelines) (40, 44), lower and upper limb (3 guidelines) (20, 34, 44) and thromboembolism (2 guidelines) (31, 44, 83). Topics in sexuality (32, 44, 51) and psychological issues (23, 38, 44, 49) were also addressed by different guidelines. A total of two guidelines cover the specific issues around osteoporosis (21, 44) and pregnancy in women with SCI (17). Furthermore, one Cochrane review does not focus on medical issues but describes the use of automated telephone communication systems in the context of prevention and management of long-term condition (43, 44).

The Guideline for Care of People with spina bifida from the American Spina Bifida Association is very comprehensive, covering a wide range of topics related to health problems and self-management (44). The sources provide information on the causes, symptoms, diagnosis, and treatment of spina bifida. The guideline is informed by current self-management research for people with spina bifida and offers recommendations to promote self-management and independence across the lifespan. The guideline covers 25 areas of physical health, mental health, and general well-being for people with spina bifida, from birth to adulthood.

Most of the included guidelines were developed and published in the United States of America (14), and Germany (9), while others were published in the United Kingdom (5) or Canada (2). The remaining guidelines were developed within global or European committees (3).

### 3.6 Methodological appraisal of the guidelines and publications

#### 3.6.1 DELBI—guidelines

A total of 34 guidelines were included in the study. All guidelines were methodologically assessed by the DELBI tool (54) and the results are summarized in Supplementary Table S5. Most guidelines perform very well in terms of formulating clear objectives regarding scope and purpose. Additionally, most guidelines demonstrate editorial independence. However, guidelines tend to perform poorly in terms of integrating and involving interest groups, as well as in methodological accuracy during the development process. Similarly, guidelines tend to receive a poor rating in terms of applicability in the German healthcare system. This remark refers specifically to the German healthcare system and cannot be generalized globally.

#### 3.6.2 R-AMSTAR—systematic reviews

Three systematic reviews (59, 69, 75) were identified in the search results and their quality were assessed with the R-AMSTAR tool (53). The overall quality of the studies was assessed as low quality. Not fully addressed were especially quality items related to the methodological reporting. The results of the quality assessment of all included systematic reviews are summarized in Supplementary Table S6.

#### 3.6.3 STROBE—observational studies

Total quality scores ranged from 9 (73) to 19 points (62). None of the 13 observational studies full field all quality standards set by STROBE (52). In particular, the quality criteria were not met in the methods and results sections (e.g., no effort to address potential sources of bias). The results of the quality assessment of all included observational studies are summarized in Supplementary Table S7.

#### 3.6.4 JBI—randomized controlled trials

A total of four randomized trials (61, 72, 74, 79) were included in the search and rated by JBI critical appraisal checklist (55). None of the trials included met all the 13 quality criteria. The main issues identified are lack of blinding and inappropriate analysis of group differences. The results of the quality assessment of the four included randomized controlled trials are summarized in Supplementary Table S8.

## 4 Discussion

The current comprehensive and extensive literature review sought to summarize recommendations on follow-up care in SCI/D and spina bifida. The search reveals only a limited number of publications of follow-up care programs regarding content, frequency and setting of follow-up programs. None of the guidelines enhances recommendations on a comprehensive medical follow-up including all relevant health conditions for people with SCI/D. There is still a lack of high-quality studies and comprehensive guidelines on follow-up care including recommendations on content, frequency and setting. This is surprising, most individuals with SCI/D may not only benefit from follow-up care at outpatient rehabilitation centers, with regard to optimizing ongoing recovery and adjustment to life with SCI/D, but it is also related to better survival (12).

### 4.1 Recommendations on content of follow-up care

The current retrieved publications cover though a broad range of different health conditions, including urological problems, pain, pressure injuries and osteoporosis (48, 58–62). Although clinical opinion is to perform comprehensive follow-up care in SCI/D and spina bifida, we found hardly any publications or guidelines describing this comprehensive approach and covering all relevant health conditions for persons with SCI/D or spina bifida. Theoretically, with the ICF generic core set and the ICF Core set for spinal cord injury in the long-term context, an ideal framework for the development of an evidence based comprehensive clinical practice guideline for follow-up care would exist and the current findings of this review could serve as an evidence-based basis for a comprehensive clinical practice guideline as commissioned by the DMGP.

### 4.2 Recommendations of setting of follow-up care

The current review served as a framework and a basis for the development of a guideline for long-term follow-up care in persons with SCI/D. This guideline gives recommendations on frequency, setting and content of follow-up care (with specific recommendations for persons with tetraplegia, spina bifida, and elderly). Follow-up care of persons with SCI/D encompasses much more as prevention and early treatment of SHCs. Besides the assessment and evaluation of body structures and body functions, regular evaluation and assessment of activities, participation, environmental factors, personal factors, and quality of life should be performed during each follow-up visit. The ICF core sets build a framework for the evaluation of all relevant aspects of functioning with SCI. The current review shows that various health care specialists are involved in the prevention and early diagnosis of SHCs [among which, general practitioner, Physical Medicine and Rehabilitation (PMR), neurologist, neuro-urologist, physiotherapy, occupational Therapy etc.] and coordination of care is complex. From earlier studies we know that persons with SCI/D frequently contact their general practitioner for a SCI related health care issues. The guideline recommends specialist in SCI care (ideally a PMR specialist) to be the coordinator of follow-up care, in close collaboration with all included health care specialist, especially with the general practitioner. The guideline therefore is not only a tool for SCI specialists, but also informs general practitioners and persons with SCI on the recommendations for follow-up care. Most publications and guidelines highlighted the multidisciplinary approach to care, with a focus on the coordination of medical services by a SCI/D specialist. This approach would involve rehabilitation physicians assessing patients and determining whether they require more specialized care, such as orthopedic or neurological care. By acting as gatekeepers, as suggested by Veenboer et al. (77), rehabilitation physicians could help ensure that patients receive the appropriate care in a timely and efficient manner. Across the continuum of care, especially for older and less mobile persons (84) regular visits at the specialized center might be difficult. Innovative approaches such as telemedicine and local visits by nurses might help to ensure specialized care in those vulnerable population. This approach could also help to reduce healthcare costs by preventing unnecessary referrals to specialists.

Bakketun et al. (80) conducted a retrospective cohort study to investigate the healthcare setting in which consultations took place for patients with MMC. These findings suggest that outpatient consultations are more common for patients with MMC, with gastroenterology being the most frequently consulted specialty. However, when persons with MMC require inpatient care, medical issues in neurosurgery are the most common reason for admission. These results highlight the importance of appropriate healthcare setting for persons with MMC, and the need for effective communication and coordination between specialties to ensure optimal care. Further research is needed to investigate the reasons for the high admission rate in medical issues in neurosurgery and to develop strategies to reduce the need for inpatient care in this population. In rural or underserved areas can be challenging due to various factors. However, telemedicine or collaboration with primary care providers may help to address these barriers and ensure that individuals with SCI/D receive best possible care and support (71, 74).

### 4.3 Recommendations on frequency of follow-up care

Although many of the retrieved publications highlight the importance of lifelong care, to prevent and manage SHCs, they do not communicate specific recommendations about frequency of follow-up care. The frequency of follow-up care may vary based on the severity of the SCI and the individual's needs (9, 58, 59). Given the large number and diversity of newly emerging problems, regular and multidisciplinary surveillance of people with SCI/D is recommended (69, 77).

Veenboer et al. (77) provide a recommendation for the frequency of follow-up care for patients with MMC. The authors suggest that regular visits to an outpatient clinic should occur every 18–24 months. This recommendation is based on the authors’ clinical experience and expertise in the management of MMC patients. The frequency of follow-up care for MMC patients is an important consideration, as it can impact patient outcomes and healthcare costs. However, there is limited research on the optimal frequency of follow-up care for MMC patients. Only one paper was found in the search results that provided a recommendation for the frequency of follow-up care for persons with MMC. This highlights the need for further research in this area to develop evidence-based guidelines for the management of MMC patients. In the meantime, healthcare providers should consider the recommendation provided by Veenboer et al. (77) when determining the frequency of follow-up care for MMC patients.

The search results for persons with spina bifida highlight the importance of understanding their health needs, particularly as they transition into adulthood. The articles provide valuable insights into the management of spina bifida, including rehabilitation, urological management, and medical and psychosocial problems. The findings can inform the development of evidence-based practices in healthcare to improve outcomes for individuals with spina bifida.

### 4.4 Methodological quality

Considering the methodological quality of included publications, we can conclude that high quality recommendations for follow-up care for people with SCI/D and spina bifida are largely missing. With the application of the STROBE tool for observational studies, the AMSTAR checklist for systematic reviews, the DELBI Tool for guidelines, and the JBI-tool for randomized Trials we provide a valuable baseline to inform the development of an evidence-based practice guideline in follow-up car for people with SCI/D. As there were also publications with methodological limitations included, there might be a risk of bias considering the reliability of specific recommendations for follow-up care for individuals with SCI/D and spina bifida.

The results of this systematic review may be limited by the following factors: The search was performed in 2019 as a first step of the development of the guideline on follow-up care of secondary health conditions in spinal cord injury and spina bifida” and thus included literature from January 2008 until December 2018. It might be that after this period, relevant articles and guidelines might have been published, which we have not included in the current review. A revision of the guideline for follow-up care, including an update of the review is planned and due on 1.1.2027 and will include all new literature and guidelines published.

We conducted our systematic search in PubMed, Cochrane library and in several Guideline databases, nevertheless there is a residual probability that a publication on this topic has not been included. Also, regarding the language, where only German and English were considered. Additionally, 12 out of the 19 publications (in PubMed) were conducted in Canada, Australia, or the United States of America (USA). Three were conducted in Switzerland and two in Germany. The other publications were from Bangladesh, Belgium, Italy, or England. The high number of English-speaking countries might be due to the lack of translation of publications in other languages. Most publications come from the USA which can partly be explained due to the reasonably shorter initial rehabilitation period for people with SCI and therefore, the more important out-patient phase. There is an underrepresentation of evidence on long-term follow-up care in low and middle income countries.

The current review of literature and guidelines also clearly showed us research gaps, for example the lack of evaluation of existing health care provision. For example, although telerehabilitation might be an upcoming service provision for patients with SCI/D, research on this topic is still a relative unexplored field. We found a limited number of publications on telerehabilitation in very diverse health care settings. The primary reason for not conducting a meta-analysis were the limited number of publications and the high degree of heterogeneity among the included studies in terms of population, interventions, and outcome measures. Given this variability, pooling the results would not have been appropriate or informative. To effectively and efficiently meet current and future challenges, an infrastructure and culture are needed where the best evidence is systematically made available and used, and the system evolves on the basis of a constant exchange between research, policy, and practice. This review is start of the development of a clinical practice guideline on follow-up care in persons with SCI and spina bifida and as such part of a Learning Health System (LHS) ensuring continuous improvement through ongoing research and implementation (85). The idea of a LHS assumes that a health system can learn when it can rely on cyclic processes where data for the health system serve as a basis for the generation of new evidence. Especially for complex conditions, such as SCI/D or spina bifida, establishment of an LHS is helpful to ensure evidence to be integrated in clinical practice and experience from clinical practice to be integrated in new research.

Finally, follow-up care of persons with SCI encompasses much more as prevention and early treatment of SHCs. Besides the assessment and evaluation of body structures and body functions, regular evaluation and assessment of activities, participation, environmental factors, personal factors, and quality of life should be performed during each follow-up visit. The ICF and its core sets build a framework for the evaluation of all relevant aspects of functioning with SCI. Although highly relevant, it was beyond the scope of the current guideline to describe all aspects of follow-up care in persons with SCI.

Conclusion: Based on the comprehensive and extensive literature research conducted, we recommend regular (annual) follow-up care appointments at specialized SCI clinics. While several specific health concerns (SHCs) were addressed in follow-up care programs, including pressure injuries, pain, and bowel issues, there is a notable absence of a comprehensive clinical practice guideline (CPG) covering all health conditions relevant for the long-term follow-up of individuals with SCI/D or spina bifida. In response to this gap, the DMGP has commissioned its members to establish a guideline for follow-up care for individuals with SCI/D. The current review serves as an evidence-based framework for the development of this guideline.

## Data Availability

The original contributions presented in the study are included in the article/Supplementary Material, further inquiries can be directed to the corresponding author.
