# Supplementary material for: Recommendations for long-term follow-up care of secondary health conditions in spinal cord injury/disorder: a systematic review
Source: Front Rehabil Sci. 2024 Oct 11;5:1371553. doi: 10.3389/fresc.2024.1371553 (PMC11502465; doi:10.3389/fresc.2024.1371553)
Supplement: Supplementary file 1 [file Table1.docx]

Supplementary Material

# Supplementary Tables

**Table 1: Search strategy in PubMed for SCI/D and Spina bifida**

|  | **Search strategy for SCI/D** |
| --- | --- |
| #1 | spinal cord injuries (mh) OR spinal cord disease (mh) OR paraplegia (mh) OR quadriplegia (mh) OR spinal injuries (mh) OR tetraplegia [tiab] OR myelopathy [tiab] OR myelopathies [tiab] OR spinal cord disorder* [tiab] OR spinal cord injury [tiab] |
| #2 | Aftercare [mh] OR ambulatory care [mh] OR long-term-care [mh] OR outpatient clinic [mh] OR disease management [mh] OR follow-up care [tiab] OR continuity of patient care [tiab] OR outpatient care [tiab] OR outpatient services [tiab] OR follow-up visit [tiab] OR follow-up program [tiab] OR follow-up service [tiab] OR follow-up intervention [tiab] OR follow-up system [tiab] OR outpatient facility [tiab] OR ambulatory facilities [tiab] OR community care [tiab] OR prevention program [tiab] OR prevention [tiab] |
| #3 | epidemiology [mh] OR morbidity [morbidity] OR health [health] OR mortality [mh] OR death [mh] OR life expectancy [mh] OR clinical effectiveness [tiab] OR health problems [tiab] OR health conditions [tiab] OR secondary conditions [tiab] OR secondary impairments [tiab] OR complications [tiab] OR secondary complications [tiab] OR secondary health conditions [tiab] OR medical problems [tiab] OR long-term medical complications [tiab] OR long term outcome [tiab] OR clinical outcome [tiab] OR health status [mh] OR rehospitalization [mh] OR readmission [mh] OR hospital readmission [mh] |
| #4 | #1 AND #2 AND #3 |
|  | **Search strategy for Spina bifida** |
| #5 | Spina Bifida [tib] OR meningomyelocele [mh] |
| #6 | #5 AND #2 AND #3 |
|  |  |
|  | * Filters: Humans, English, German, from 2008/1/1 – 2018/31/12 |

**Table 2: Search strategies in different guideline databases**

| **Database** | **Search term/ -strategy** |
| --- | --- |
| AWMF | Search 1: Querschnittlähmung |
|  | Search 2: Spina bifida |
| SCIRE | All |
| NICE | Search 1: Spinal cord injury |
|  | Search 2: Spina bifida |
| NGC | Search 1: Spinal cord injury (Exact Phrase: Guideline Spinal cord) |
|  | Search 2: Spina bifida (Exact Phrase: Guideline) |
| Guidelines British Society of Rehabilitation Medicine | Search 1: Spinal cord injury |
|  | Search 2: Spina bifida |
| Clinical practice guidelines for persons with spinal cord injury | Search 1: Alle |
|  | Search 2: Spina bifida |
| Cochrane | Search 1: Spinal cord injury/disorder/dysfunction |
|  | Search 2: Spina bifida/Meningomyelocèle |
| GIN | Search 1: Spinal cord injury/injuries/dysfunction |
|  | Search 2: Spina bifida/Meningomyelocèle |
| American Spina Bifida Association | All |
|  |  |
| * Filters: English, German, up to 2018/31//12 |  |

Table 3: Follow-up care characteristics for publications in SCI/D

|  | **Author** | **Aim** | **Method / Design** | **Content / Recommendations** | **Frequency and setting of follow-up care** |
| --- | --- | --- | --- | --- | --- |
| 1 | Przydacz et al. 2018 [1] | Recommendations for urological follow-up of patients with neurogenic bladder secondary to spinal cord injury | Review  Research in PubMed, Cochrane Library, and Web of Science. | Different surveillance strategies exist, but there is no consensus among authors and organizations. As a result, practice patterns vary around the world. Urological follow-up of SCI patients should consist of medical history (LE 1-4, GR B-C), clinical examination (LE 4, GR C), renal laboratory tests (LE 1-3, GR B), imaging surveillance of the upper urinary tract (LE 1-3, GR A-B), urodynamic study (LE 2-4, GR B-C), and cystoscopy/cytology (LE 1-4, GR D). | 1- year follow-up schedule for patients without additional risk factors of renal deterioration.  Modified follow-up plans with more frequent check-ups for those who manifest risk factors (individually tailored approach) |
| 2 | Mc Coll et al. 2017 [2] | Asisst family physicians to play their role in promoting the health of people with SCI, by summarizing the latest evidence in the management of SCI in primary care | Scoping Review  Research in Pubmed, OVID, CINAHL and published in English. | (1) Pharmacological management of pain (2) Neurogenic bowel: Patients with persistent constipation should be referred to a specialist with experience in SCI (3) Screening for colon cancer: same principles as for the general population (4) Detection and diagnosis of urinary tract infections (UTI) (5) Screening for bladder cancer: Routine screening for bladder cancer is only recommended for high risk patients i.e. those with permanent or suprapubic catheters, complete lesions, <10 years after injury, bladder stones or recurrent urinary tract infections. (6) Treatment of skin lesions (7) Wheelchair accessibility of your practice (8) Screening for cardiovascular risk and management of cardiovascular risk (9) Autonomic dysreflexia (10) Management of musculoskeletal pain (11) Neurogenic bowel (12) Evaluation of the bowel management program (13) Neurogenic bladder (14) Prevention of skin rupture (15) Depression and paraplegia (16) sexuality in SCI | - Pharmacological management of neuropathic pain: annually  - Neurogenic bowel: annually  - Bowel Management: periodic Re-evaluation  - Neurogenic bladder: annually by family physician and periodically by a urologist  - Cardiovascular risk factors: at least annually  - Depression: Screen annually  - Sexuality: annual examination |
| 3 | Gemperli et al. 2017 [3] | Identify the care-seeking behavior of persons with SCI with respect to the various health care providers | Statistical analysis of frequency of annual visits to health care providers by 17 specialties, and description of situations where health care was required but not received | The main contact person was the family doctor (with around 88% of patients having had an appointment in the past 12 months). Persons with paraplegia are frequent users of medical services. The most frequently mentioned health problems for which health care support was required, but not received or sought, were bladder problems (47%) followed by bowel problems (32%) and medication needs (25%). However, no specialist covers all the needs of paraplegic patients, which is why it makes sense to offer a broad range of medical services. | No recommendations on frequency |
| 4 | Mitchell et al. 2017 [4] | Describe the use of health services before and after the index of injuries over a period of 12 months (by injury type and injury severity). | Population-based matched-cohort study for hospital and mortality rates in Australia.  Total sample size:  N (injured) = 167'600  N (non-injured) = 167'600 | The injury cohort experienced higher 12-month hospital admissions before and after the injury than the non-injured group. After 6 to 7 months after injury, the injury cohort had largely returned to pre-injury hospital admission levels, except for injuries involving dislocation, sprains and strains, and injuries to nerves and spinal cord.  Hip fractures (17.69 per 100 person months) and poisoning (16.09 per 100 person months) had the highest rates of hospitalisation after injury in the Injury Cohort. The adjusted rate ratios (ARR) for hospital stays after injuries were highest for poisonings (ARR: 3.77; 95% CI: 3.38-4.21) and injuries of nerves and spinal cord (ARR: 2.73; 95% CI: 2.27-3.299).  Many people are returned to hospital after a traumatic injury and require continuous care for up to 12 months after injury. | Seriously injured trauma patients should be followed for at least two years post-injury regarding their post-injury health outcome. Thus, trauma services should consider long-term aftercare and support services for critically injured patients after discharge from hospital. |
| 5 | Böthig et al. 2017 [5] | Recommendations for diagnosis and therapy of neurogenic bladder dysfunction | Consensus-based guideline according to the AWMF criteria (S2k) | The guideline sets out the principles and objectives of neurourological care of patients with SCI and discusses in detail the principles of diagnosis and therapy of neurogenic bladder dysfunction. The necessity of video-urodynamic examinations as a basis for the classification of neurogenic bladder dysfunction and as a basis for the development of a treatment strategy is emphasized. Both conservative and surgical-operative treatment options and their indications are explained in detail. Possible complications and their prevention in the long-term course after SCI are presented with special consideration of the specifics of urinary tract infections and autonomic dysreflexia. | Lifelong individual and risk-based care is needed with specific recommendations on diagnostics and therapy for neurourological problems |
| 6 | Hossain et al. 2017 [6] | Explore the feasibility of conducting a full trial designed to determine the effectiveness of a model of community-based care for people with SCI in Bangladesh | Pilot randomized trial of 30 people with recent paraplegia who were wheelchair dependent and will soon be discharged from hospital.  Intervention group: care package with regular telephone contact and three home visits over two years. Control group: usual care package consisting of a telephone call and an optional home visit. | The primary endpoint: mortality, secondary endpoints: complications, depression, participation and quality of life. A total of 24 participants had a complete spinal cord injury and six participants had an incomplete spinal cord injury. The median (interquartile) age and time since injury at baseline were 31 years (24 to 36) and 7 months (4 to 13), respectively. Two participants, one in each group, died. Five participants had pressure ulcers at the age of two years. There were no significant disabilities in the conduct of the study and no significant protocol violations. Telephone calls and home visits were conducted in accordance with protocol in 87% and 100% of cases, respectively. The follow-up data were 99% complete. | No recommendations on frequency, but in general challenging to provide regular follow-up in low- and middle-income countries |
| 7 | Heinemann et al. 2016 [7] | To evaluate the perception of person-centred care (PCC) in persons with traumatic SCI; and (2) to investigate the perceived differences in PCC concepts between patients who continue to receive services from a Spinal Cord Injury Model System (SCIMS) facility and those who do not | Cross-sectional design  Outcome measures: Participants completed the Patient Activation Measure (PAM), the Patient Assessment of Chronic Illness Care, the Global Practice Experience Measurement and 5 Press-Ganey questions to assess the key elements of patient- and family-centered care. | The patient perspective on the care of the chronically ill was more positive among SCIMS users than among non-users (3.15 vs. 2.91, P<.05); the difference is primarily due to higher subscale values in targeting and adjustment. SCIMS users and non-users did not differ in PAM total score or activation stage, global practice experience, or Press Ganey scores.  Conclusion:  SCIMS users and non-users differed in their perception of the treatment of chronic diseases (mainly in the targeting and adaptation component), which was more favorable for the users of the model system than for non-users. The results can serve as a guideline for strategies to improve PCC practice after inpatient rehabilitation. | No recommendations on follow-up |
| 8 | Van de Pol et al. 2015 [8] | Review of ambulatory services provided by the QSCIS (Spinal Injuries Unit = SIU and Spinal Outreach Team = SPOT) in Queensland to help identify where telehealth may potentially be useful. | Cohort study -  The services provided by the SIU outpatient clinics and SPOT over a period of 6 years (2008- 2013) were analysed. The general population of SCI patients was compared with patients treated by SIU outpatient clinics and the SPOT. | During the 6-year period, 2073 patients were referred to QSCIS (and were alive at the time of analysis). 74% of all patients were male. The median age was 51 years (IQR39y-61y). About two thirds of all patients lived within 200 km of Brisbane. 24% of all patients registered with QSCIS lived more than 200 km from Brisbane. 7513 appointments were made in the SIU outpatient clinic. At selected regional locations, 43827 service appointments were held for up to 100 patients per year. 13 video conference appointments were replayed. 90% of all patients who visited the SIU Outpatient Clinic lived within 200 km of Brisbane.  Since one-third of all patients registered with QSCIS live at least 200 km from Brisbane, these patients may not be able to use the same services as patients resident in Brisbane. Telemedicine care models that encourage better collaboration with local healthcare providers could improve equity of access and reduce the need for extended travel. | No recommendation on frequency, but Importance of ongoing and lifelong management and rehabilitation or people with SCI. Additional recommendation for telehealth methodologies especially for people at the distant sites. |
| 9 | Saur & Abel, 2015 [9] | Description of SCI rehabilitation and most relevant aspects for SCI | Opinion paper | For long-term follow-up main aspects in orthopedic aspects: development of a scoliosis, contractures, pressure ulcer) neurological aspects (spasticity, pain, syringomyelia), urological aspects (Bladder-Bowel- sexual function) and internal aspects. | No specific recommendation but importance of comprehensive care and lifelong follow-up of paraplegic patients |
| 10 | Spreyermann & Michel, 2014 [10] | Recommendations for prevention and follow-up for people with SCI | Guideline | Regularly ambulatory long term follow-up visits in specialized centers in close collaboration with general practitioners help to diminish complications and rehospitalizations. Facing the now ageing population with a SCI there is evidence-based guidelines needed in follow-up and preventive strategies for these patients. The authors updated these recommendations recently. | Annual check-up of: (1) Neurology, (2) orthopedic aspects, (3) Bladder, bowel and sexual function, (4) social and occupational situation aspects, (5) Prevention (e.g. ultrasound of bladder and kidney, lipid profile, lung function), (6) internal medicine (e.g. vaccination, cancer prevention) |
| 11 | Stiens et al., 2013 [11] | Explication of all qualifications required of life care planners to project the needs of person with SCI and the full methodology to develop a life care plan for patients with SCI. | Opinion paper  Problem-oriented chart review | Complications and concerns for SCI patients:  1. nervous system: a. chronic pain, neurogenic pain, b. post-traumatic cystic meyelopathy, c. post-traumatic syringomyelia, d. spasticity, e. fatigue and weakness 2. respiratory system: a. pulmonary infections, b. pumonary function, c. reduced vital capacity 3. musculoskeletal system: a. Restriction of the upper extremities, b. Degenerative changes, c. Fractures, d. Osteoporosis, e. Postural defects, f. Contractures, g. heterotrophic ossification 4. cardiovascular system, a. autonomic dysreflexia b. deep vein thrombosis, c. coronary heart disease 5. gastro-enteral system: a. abdominal pain, abdominal distension, b. gastro-intestinal bleeding, c. gastric ulcer, d. intestinal obstruction, e. oesophageal problems, g. Diverticula, h. Hemorrhoids, i. Bowel movement, j. constipation, k. diarrhoea, l. delayed bowel movement, m. faecal continence 6. urogenital system: a. urinary tract infection, b. bladder cancer, c. kidney infection, kidney stones, e. urethral tears f. hydronephrosis, g. Bladder dilatation, h. Bladder trabeculation, i. Chronic cystitis, j. prostate, k. epidimyorchidis7. skin: a. pressure points 8. psychosocial function: a. Adaptation, b. Adaptation: i. Apathy, ii. Anxiety, iii. Depression, iv. Social isolation, v. suicidal thoughts, c. self-control, d. substance abuse | Type of periodic evaluations and its frequency are determined by the level of injury, time after injury, and other associated diagnoses (such as traumatic brain injury and risk factors for coronary artery disease). |
| 12 | Guilcher et al., 2013 [12] | Understand the journey of care in the prevention and management of SHCs following SCI | Case-study design (Qualitative research paper)  The Network Episode Model was used as the conceptual framework. Data sources included in depth interviews with persons with SCI, care providers, and policy and decision makers. | A major over-arching domain that emerged from the data was the concept of 'fighting'. Findings suggest that the journey is challenging a persistent uphill struggle for persons with SCI, care providers, and community-based advocates. If we are to make significant gains in minimizing the incidence and severity of SHCs, we need to tailor efforts at the health system level.  The lack of rehabilitation services available within the publicly funded system once persons are discharged to the community was highlighted as a key barrier in preventing and managing SHCs. | This study stresses the importance of timely access to comprehensive primary health care. |
| 13 | Young-Hughes & Simbartl, 2011 [13] | Compare costs of providing specialty wound care to spinal cord injury/disorder (SCI/D) veterans by teleconsultation and traditional care | A retrospective design was used to conduct this descriptive, correlational study. A convenience sample of 76 SCI/D veterans (2 women, 74 men) met inclusion criteria from a possible 123 subjects. | There was no significant difference in inpatient admissions or inpatient bed days of care between the two groups. The teleconsultation group had more outpatient encounters (medians 12 vs. 4, p = .007; Wilcoxon statistic = 412.5) and longer inpatient stays (medians 81 vs. 19 days / admission, p = .05; Wilcoxon statistic = 227.0) compared to the traditional care group. There was no significant difference in inpatient cost between the two groups; however, the teleconsultation group had a significantly higher median cost per outpatient encounter ($440 vs. $141, p <.0001; Wilcoxon statistic = 469.0). Although this study only looked at costs directly associated with wound management, continued research exploring the use of teleconsultation in other areas of SCI/D specialty is needed to enhance its application. | No recommendations about frequency, but stresses the importance of wound care and prevention for people with SCI/D |
| 14 | Spreyermann et al., 2011 [14] | Create a new electronic tool based on the ICF that enables information to be registered and visualized, including the use of a net-diagram to show a patient's long-term development | Study design: develop a computer program that supports the overview of a follow-up care process in people with SCI in daily clinical practice | Outpatient setting, four perspectives were assessed: patient, physician, occupational therapist and physiotherapist for a comprehensive bio-psycho-social consideration. All categories were assessed and graphically visualized with the electronic tool, based on the ICF domains. The tool facilitates the patient counselling and the interdisciplinary work in daily clinical practice. Such a visual report helps to recognize predispositions over time. Furthermore, it helps to explain the clinical and patient-related findings accessible to the patients, to involve them as participants in defining the goals and the treatment plan. | Annual check-up based on the patient's answer in the electronical ICF-tool |
| 15 | Careau et al., 2010 [15] | The aim of the study was to document the workings of an interprofessional rehabilitation team videoconference and the advantages and disadvantages of this method. | Observational study: 11 ICP meetings for clients with SCI on DVD were recorded to get simultaneous interaction from both groups (specialized and regional center). | The high rate of productivity informs us that videoconferencing is an effective strategy for conducting ICP meetings. The client had the highest participation rate, showing an active role in the development of his/ her own care plan. | No recommendations about frequency or content of follow-up |
| 16 | Morse et al., 2009 [16] | This study sought to characterize the diagnosis and management practices within the VA health care system for osteoporosis following SCI. | Online survey regarding osteoporosis management in SCI composed of 27 questions designed to gather information on responder demographics, osteoporosis diagnostics, and treatment options. | Ninety-two prescribing practitioners (physicians, nurse practitioners, and physician assistants) were included in the analysis. Of these respondents, 50 (54%) prescribe medications for SCI-induced bone loss; 39 (42%) prescribe bisphosphonates and 46 (50%) prescribe vitamin D. There were 54 (59%) respondents who routinely order diagnostic tests, including dual energy x-ray absorptiometry scans in 50 (54%). Variations in practice were not explained by age, gender, or years practicing SCI medicine. Many respondents (23%) reported barriers to osteoporosis testing including lack of scanning protocols, cost, wheelchair inaccessibility of scanning facilities, and lack of effective treatment guidelines once osteoporosis is diagnosed. | Despite an absence of screening and treatment guidelines, more than half of all respondents are actively diagnosing and treating osteoporosis with bisphosphonates within the VA health care setting. These data suggest that evidence-based practice guidelines are necessary to reduce practice variations and improve clinical care for this population. |
| 17 | Dallolio et al., 2008 [17] | To compare the 6-month outcomes of telerehabilitation intervention with those of standard care for spinal cord injury (SCI). | Multicenter randomized controlled trial.  PARTICIPANTS: Adult patients with nonprogressive, complete, or incomplete SCI discharged for the first time from the spinal cord unit to their homes (Belgium and Italy) or to their homes or another facility (England).  INTERVENTIONS: Patients in the telemedicine group received 8 telemedicine weekly sessions in the first 2 months (additional to the standard care), followed by biweekly telemedicine sessions for 4 months.  OUTCOME MEASURES: Functional status at 6 months, clinical complications during the postdischarge period, and patient satisfaction. | No significant differences in the occurrence of clinical complications were found between the study groups. A higher improvement of functional scores in the telemedicine group was found only at the Italian site: FIM total score 3.38+/-4.43 (controls) versus 7.69+/-6.88 (telemedicine group), FIM motor score 3.24+/-4.38 (controls) versus 7.55+/-7.00 (telemedicine group; P<.05). Items contributing to this difference were grooming, dressing upper body, dressing lower body, and bed/chair/wheelchair transfer. Higher satisfaction with care was reported by patients in the telemedicine group across all sites.  Our study provides some of the first quantitative evidence, based on results from 1 site, that telerehabilitation may offer benefits to patients discharged from a spinal cord unit compared with standard care in terms of functional improvement. Further research is warranted to confirm or disprove this finding. | No recommendation on frequency and content of follow up care. Telemedicine as an option for |
| 18 | Bendixen et al., 2008 [18] | This article provides an overview of telehealth, explains the LAMP model, and presents a case history of a veteran who sustained complete tetraplegia and traumatic transfemoral amputation as the result of a blast injury and who lives successfully at home with the support of LAMP. | Overview and cost analysis  The Low Activities of Daily Living Monitoring Program (LAMP) at the North Florida/South Georgia. Initially designed to serve elders at risk of institutionalization, LAMP now is being adapted to the needs of veterans living with the effects of multisystem polytrauma.  Total sample size: N = 230 (NLAMP: 115, Matched comparison group: 115) | A recent cost analysis of LAMP patients compared to a matched cohort receiving standard care also is presented. The LAMP model shows promise as a method for home-based management of combat-wounded veterans who experience multisystem polytrauma.  Although the complexity of care required by many of these veterans seems ideally suited for a care coordination and home-based TRH (Telerehabilitation) intervention, the approach may need to be broadened to include an interdisciplinary team of CCs (Care coordinators). | No recommendation on frequency |
| 19 | Rintala et al., 2008 [19] | To test the hypothesis that enhanced education and structured follow-up after pressure ulcer surgery will result in fewer recurrences. | Randomized controlled trial  SETTING: Veterans Affairs medical center. 49 men with SCI. Group 1 received individualized pressure ulcer education and monthly structured telephone follow-up (n=20); group 2 received monthly mail or telephone follow-up without educational content (n=11); and group 3 received quarterly mail or telephone follow-up without educational content (n=10). Follow-up continued until recurrence, death, or 24 months. Time to pressure ulcer recurrence. | Group 1 had a longer average time to ulcer recurrence or end of study than groups 2 and 3 (19.6 mo, 10.1 mo, 10.3 mo; P=.002) and had a smaller rate of recurrence (33%, 60%, 90%; P=.007). Survival analysis confirmed these findings (P=.009). | Individualized education and structured monthly contacts may be effective in reducing the frequency of or delaying pressure ulcer recurrence after surgical repair of an ulcer |

**Table 4: Follow-up care characteristics for publications in spina bifida**

|  | **Author** | **Aim** | **Method / Design** | **Content / Recommendations** | **Frequency and setting of follow-up care** |
| --- | --- | --- | --- | --- | --- |
| **1** | Bakketun et al. 2019) [20] | Evaluate correlation between childhood health condition related to MMC and the need for specialized consultations in adulthood | Retrospective cohort study on adults (n=38 patients with 372 consultations) with MMC | 38 Patients had 672 consultations in the hospital related to MMC, due to various health problems | Patients need life-long multispecialized follow-up and rehabilitation  Majority of consultations were in outpatient setting (e.g. gastroenterology 83%). Highest rate for inpatient setting were medical issues in neurosurgery 36%. |
| **2** | Barker et al. (2017) [21] | Evaluate the impact of a person-centered, community rehabilitation service on outcomes for people with a neurological condition, in the first year of service | Prospective observational pre-post study (n=206, not only spina bifida) | Participants demonstrated significant goal achievement and a significant Reduction in health and medical resource use (as outcome of a person-centered community rehabilitation). | No recommendation on frequency  Provide person-centred community rehabilitation (in the first year of service) tailored to the unique lifestyles of individuals with neurological conditions |
| **3** | Dicianno et al. (2008) [22] | Summarizing current evidence-based practice, and identifies key areas in which scientific evidence is lacking and future research is needed | Literature review of literature 1988-2008 on rehabilitation care for spina bifida | As medical treatment continues to improve for individuals with SB, they will continue to live longer and will encounter a wide range of medical, rehabilitation, and psychosocial complications. Optimal management of these complications would allow adult individuals with SB to maximize their function and quality of life. | Optimal and lifelong management of medical complications |
| **4** | Khan et al. (2015) [23] | Assess s the effectiveness of an interdisciplinary ambulatory Rehabilitation program for persons with spina bifida in an Australian community cohort | Randomized controlled trial. Fifty-four participants were randomized to a treatment group for high intensity ID rehabilitation program (with cognitive-behaviour therapy), or a control group comprising usual care. | Adjusted for baseline disease and demographic covariates, the intervention group improved significantly at 3-month follow-up for primary and secondary outcomes, with moderate to large effect sizes | To offer targeted rehabilitation care for maintaining activity and participation over the longer-term.  No specific recommendation about setting or frequency |
| **5** | Szymanski et al. (2015) [24] | Elucidate current practices and opinions regarding the management of adult complex genitourinary patients by pediatric Urologists, in order to determine if a consensus for adult care exists. | Anonymous 15-question online survey (sample size: 62) | Pediatric urologists appeared to be virtually unanimous in recommending that urologists provide the most appropriate long-term follow-up of Patients with congenital genitourinary conditions. | Urologist (instead of primary care physician) as the most appropriate long-term follow-up for persons with congenital genitourinary conditions.  No recommendation about frequency |
| **6** | Veenboer et al. (2014) [25] | To identify physical, emotional and psychosocial issues in the older persons with spina bifida (SB) | Survey (sample size: 61) | A majority of adult persons with SB reported newly arising physical and psychological problems during the previous 5 years. An important finding: over the years, people continue to experience new problems. | Rehabilitation physician acts as a gatekeeper for more specialist care (comparable to a general practitioner, but then specialized in persons with SB).  Regular visits at outpatient clinic ( every 18-24 months) |

**Table 5: Methodological quality of included guidelines on DELBI**

| Reference | Domain | | | | | | | |
| --- | --- | --- | --- | --- | --- | --- | --- | --- |
|  | 1 | 2 | 3 | 4 | 5 | 6 | 7 | 8 |
| Böthig et al. (2016) [26] | 10/12 | 10/16 | 16/28 | 15/16 | 8/12 | 8/8 | 23/24 | 5/20 |
| Steffen et al. (2018) [27] | 12/12 | 9/16 | 15/28 | 12/16 | 8/12 | 5/8 | 10/24 | 7/20 |
| Geng et al. (2019) [28] | 12/12 | 9/16 | 17/28 | 13/16 | 6/12 | 8/8 | 20/24 | 5/20 |
| Drzin-Schilling et al. (2016) [29] | 10/12 | 9/16 | 12/28 | 10/16 | 3/12 | 8/8 | 12/24 | 6/20 |
| Kurze et al. (2018) [30] | 12/12 | 13/16 | 18/28 | 15/16 | 8/12 | 8/8 | 12/24 | 7/20 |
| Biglari et al. (2017) [31] | 8/12 | 9/16 | 14/28 | 11/16 | 7/12 | 8/8 | 11/24 | 5/20 |
| Scheel-Sailer et al. (2018) [32] | 12/12 | 11/16 | 24/28 | 12/16 | 6/12 | 8/8 | 17/24 | 14/20 |
| Kalke et al. (2018) [33] | 11/12 | 6/16 | 13/28 | 11/16 | 5/12 | 8/8 | 12/24 | 5/20 |
| Stein et al. (2013) (*) [34] | 12/12 | 11/16 | 18/28 | 14/16 | 10/12 | 8/8 | 19/24 | 6/20 |
| SCIRE Professional (2010) [35] | 12/12 | 9/16 | 17/28 | 14/16 | 6/12 | 4/8 | 14/24 | 14/20 |
| NICE (2012) (/*) [36] | 12/12 | 10/16 | 22/28 | 14/16 | 12/12 | 3/8 | 22/24 | 18/20 |
| NICE (2007) (/*) [37] | 12/12 | 10/16 | 28/28 | 15/16 | 8/12 | 6/8 | 19/24 | 18/20 |
| NICE (2014) [38] | 12/12 | 9/16 | 24/28 | 16/16 | 12/12 | 4/8 | 20/24 | 17/20 |
| British Society of rehabilitation Medicine (2009) [39] | 9/12 | 6/16 | 8/28 | 11/16 | 4/12 | 2/8 | 14/24 | 5/20 |
| Consortium for Spinal Cord Medicine, Paralyzed Veterans of America (2016) [40] | 12/12 | 11/16 | 22/28 | 16/16 | 7/12 | 4/8 | 22/24 | 12/20 |
| Consortium for Spinal Cord Medicine, Paralyzed Veterans of America (2010) [41] | 12/12 | 11/16 | 22/28 | 16/16 | 7/12 | 4/8 | 22/24 | 12/20 |
| Consortium for Spinal Cord Medicine, Paralyzed Veterans of America (2006) [42] | 12/12 | 11/16 | 22/28 | 16/16 | 7/12 | 4/8 | 22/24 | 12/20 |
| Consortium for Spinal Cord Medicine, Paralyzed Veterans of America (2005) [43] | 12/12 | 11/16 | 22/28 | 16/16 | 7/12 | 4/8 | 22/24 | 12/20 |
| Consortium for Spinal Cord Medicine, Paralyzed Veterans of America (2005) [44] | 12/12 | 11/16 | 22/28 | 16/16 | 7/12 | 4/8 | 22/24 | 12/20 |
| Consortium for Spinal Cord Medicine, Paralyzed Veterans of America (2014) [45] | 12/12 | 10/16 | 21/28 | 15/16 | 7/12 | 4/8 | 19/24 | 12/20 |
| Consortium for Spinal Cord Medicine, Paralyzed Veterans of America (1999) [46] | 12/12 | 10/16 | 21/28 | 15/16 | 7/12 | 4/8 | 19/24 | 12/20 |
| Consortium for Spinal Cord Medicine, Paralyzed Veterans of America (1998) [47] | 12/12 | 11/16 | 21/28 | 15/16 | 7/12 | 4/8 | 19/24 | 12/20 |
| Consortium for Spinal Cord Medicine, Paralyzed Veterans of America (1998) [48] | 12/12 | 11/16 | 22/28 | 15/16 | 7/12 | 4/8 | 19/24 | 12/20 |
| Nash et al. (2018) [49] | 12/12 | 11/16 | 22/28 | 13/16 | 7/12 | 4/8 | 20/24 | 12/20 |
| Coggrave et al. (2014) [50] | 12/12 | 12/16 | 28/28 | 14/16 | 7/12 | 6/8 | 16/24 | 18/20 |
| Joyce et al. (2018) [51] | 12/12 | 11/16 | 28/28 | 8/16 | 6/12 | 6/8 | 15/24 | 18/20 |
| Posadzki et al. (2016) [52] | 8/12 | 7/16 | 28/28 | 14/16 | 7/12 | 6/8 | 14/24 | 18/20 |
| Spina Bifida Association (2018) (*) [53] | 12/12 | 11/16 | 11/28 | 12/16 | 7/12 | 6/8 | 14/24 | 18/20 |
| Steffen et al. (2019) [54] | 11/12 | 8/16 | 16/28 | 14/16 | 3/12 | 8/8 | 16/24 | 5/20 |
| Schurch et al. (2018) [55] | 12/12 | 11/16 | 22/28 | 13/16 | 7/12 | 4/8 | 20/24 | 12/20 |
| Cotterill et al. (2018) [56] | 7/12 | 9/16 | 15/28 | 7/16 | 5/12 | 3/8 | 13/24 | 18/20 |
| Mehta et al. (2016) [57] | 12/12 | 8/16 | 21/28 | 13/16 | 10/12 | 2/8 | 11/24 | 5/20 |
| Russell et al. (2016) [58] | 12/12 | 12/16 | 10/28 | 10/16 | 11/12 | 2/8 | 11/24 | 5/20 |
| Albert et al. (2012) [59] | 12/12 | 8/16 | 7/28 | 9/16 | 6/12 | 5/8 | 10/24 | 5/20 |
| Wiener et al. (2018) (*) [60] | 8/12 | 4/16 | 8/28 | 6/16 | 4/12 | 2/8 | 7/24 | 5/20 |
| (*) studies for spina bifida  (/*) studies for SCI/D and spina bifida | | | | | | | | |

**Table 6: Methodological quality of included systematic reviews based on R-AMSTAR**

| Reference | Item | | | | | | | | | | | Quality of Evidence |
| --- | --- | --- | --- | --- | --- | --- | --- | --- | --- | --- | --- | --- |
|  | 1 | 2 | 3 | 4 | 5 | 6 | 7 | 8 | 9 | 10 | 11 |  |
| Przydacz et al. (2018) [1] | 1 | 1 | 2 | 2 | 1 | 2 | 1 | 1 | 1 | 1 | 2 | Low |
| McColl et al. (2017) [2] | 2 | 4 | 3 | 1 | 1 | 1 | 1 | 1 | 1 | 1 | 2 | Low |
| Dicianno et al. (2018) (*) [22] | 3 | 1 | 3 | 3 | 1 | 3 | 1 | 1 | 1 | 1 | 1 | Low |
| Based on criteria, every question should be assigned a score from 0 to 4.  (*) studies for spina bifida | | | | | | | | | | | | |

**Table 7: Methodological quality of included observational studies based on STROBE**

| Reference | Title and abstract | Introduction | Methods | Results | Discussion | Other information | Total score* |
| --- | --- | --- | --- | --- | --- | --- | --- |
| Young-Hughes & Simbartl (2011) [13] | ✓ | ✓ | ✓ | x | ✓ | ✓ | 19 |
| Guilcher et al. (2013) [12] | x | ✓ | ✓ | x | ✓ | ✓ | 18 |
| Mitchell et al. (2017) [4] | ✓ | ✓ | x | x | ✓ | ✓ | 18 |
| Heinemann et al. (2016) [7] | ✓ | ✓ | x | x | ✓ | ✓ | 16 |
| Morse et al. (2009) [16] | ✓ | ✓ | x | x | ✓ | x | 16 |
| Gemperli et al. (2017) [3] | ✓ | ✓ | x | x | ✓ | ✓ | 15 |
| Bendixen et al. (2008) [18] | ✓ | x | x | x | ✓ | ✓ | 14 |
| Van de Pol et al. (2015) [8] | x | x | x | x | ✓ | ✓ | 11 |
| Careau et al. (2010) [15] | x | x | x | x | x | x | 9 |
| Bakketun et al. (2019) (*) [20] | ✓ | ✓ | x | x | ✓ | ✓ | 17 |
| Szymanski et al. (2015) (*) [24] | ✓ | ✓ | x | x | ✓ | x | 16 |
| Barker et al. (2017) (*) [21] | ✓ | ✓ | x | x | x | ✓ | 13 |
| Veenboer et al. (2014) (*) [25] | ✓ | ✓ | x | x | x | ✓ | 13 |
| ✓: All items were adequately addressed; x: one or more items were not adequately addressed  * Total score can range from 0 to 22.  (*) studies for spina bifida | | | | | | | |

**Table 8: Methodological quality of included (non) randomized trial based on JBI tool [6, 17, 23]**

| Reference | Item | | | | | | | | | | | | | Total score* |
| --- | --- | --- | --- | --- | --- | --- | --- | --- | --- | --- | --- | --- | --- | --- |
|  | 1 | 2 | 3 | 4 | 5 | 6 | 7 | 8 | 9 | 10 | 11 | 12 | 13 |  |
| Hossain (2017) [6] | ✓ | ✓ | ✓ | ✓ | ✓ | ✓ | ✓ | ✓ | ✓ | ✓ | ✓ | ✓ | ✓ | 13 |
| Dallolio (2008) [17] | ✓ | ✓ | ✓ | x | x | x | ✓ | ✓ | ✓ | ✓ | ✓ | ✓ | ✓ | 10 |
| Rintala (2008) [19] | ✓ | ✓ | ✓ | x | x | x | ✓ | ✓ | ✓ | ✓ | ✓ | ✓ | ✓ | 10 |
| Khan (2015) (*) [23] | ✓ | ✓ | ✓ | x | ✓ | ✓ | ✓ | ✓ | ✓ | ✓ | ✓ | ✓ | ✓ | 12 |
| ✓: All items were adequately addressed; x: item was not clear or not fulfilled  * Total score can range from 0 to 13.  (*) studies for spina bifida | | | | | | | | | | | | | | |

**Table 9: Search for and evaluation of guidelines in topics of SCI/D and spina bifida**

| **Database** | **Search term / strategy** | **Total** | **Matching hits** | **Title** |
| --- | --- | --- | --- | --- |
| AWMF | Search 1: Querschnittlähmung | 18 | 8 | Neurogene Darmfunktionsstörung bei Querschnittlähmung (S2k) [28]  Depression bei Menschen mit Querschnittlähmung: Besonderheiten in der Diagnostik und Behandlung (S1) [29]  Schwangerschaft, Geburt und Wochenbett bei Frauen mit Querschnittlähmung (S2k) [30]  Schmerzen bei Querschnittlähmung (S2k) [27]  Neuro-urologische Versorgung querschnittgelähmter Patienten (S2k) [26]  Querschnittspezifische Dekubitusbehandlung und –prävention (S1) [31]  Rehabilitation der unteren Extremität, der Steh- und Gehfunktion bei Menschen mit Querschnittlähmung (S2e) [32]  Querschnittlähmungsassoziierte Osteoporose (S1) [33] |
|  | Search 2: Spina bifida | 16 | 1 | Diagnostik und Therapie der neurogenen Blasenfunktionsstörungen bei Kindern und Jugendlichen mit spinaler Dysraphie [34] |
| SCIRE | All | 27 | 1 | Rehabilitation Practices [35] |
| NICE | Search 1: Spinal cord | 15 | 3 | Urinary incontinence in neurological disease: assessment and management [36]  Faecal incontinence in adults: management [37]  Pressure ulcers: prevention and management [38] |
|  | Search 2: Spina bifida | 3 | 2 | Urinary incontinence in neurological disease: assessment and management [36]  Faecal incontinence in adults: management [37] |
| NGC | Search 1: Spinal cord injury (Exact Phrase: Guideline Spinal cord) | 3 | 0 |  |
|  | Search 2: Spina bifida (Exact Phrase: Guideline) | 1 | 0 |  |
| Guidelines British Society of Rehabilitation Medicine | Search 1: Spinal cord injury | 6 | 1 | BSRM Standards for Rehabilitation Services mapped on to the NSF for Long-term neurological conditions [39] |
|  | Search 2: Spina bifida | 0 | 0 |  |
| Clinical practice guidelines for persons with spinal cord injury | Search 1: Alle | 12  0 | 10  0 | Prevention of Venous Thromboembolism in Individuals with Spinal Cord Injury [40]    Sexuality and Reproductive Health in Adults with Spinal Cord injury [41]  Bladder Management for Adults with Spinal Cord Injury [42]  Preservation of Upper Limb Function Following Spinal Cord Injury [43]  Respiratory Management Following Spinal Cord Injury [44]  Pressure Ulcer Prevention and Treatment Following Spinal Cord Injury, 2nd edition [45]  Outcomes Following Traumatic Spinal Cord Injury [46]  Depression Following Spinal Cord Injury [47]  Neurogenic Bowel Management in Adults with Spinal Cord Injury [48]  Identification and Management of Cardiometabolic Risk [49] |
|  | Search 2: Spina bifida | 0 | 0 |  |
| Cochrane | Search 1: Spinal cord injury/disorder/dysfunction | 48/15/13 | 2/0/2 | Management of faecal incontinence and constipation in adults with central neurological diseases [50]  Organisation of health services for preventing and treating pressure ulcers [51]  Automated telephone communication systems for preventive healthcare and management of long‐term conditions [52] |
|  | Search 2: Spina bifida/Meningomyelocèle | 5/1 | 0/0 |  |
| GIN | Search 1: Spinal cord injury/injuries/dysfunction | 5 | 0 |  |
|  | Search 2: Spina bifida/Meningomyelocèle | 0 | 0 |  |
| American Spina Bifida Association | Alle | 1 | 1 | Guidelines for the Care of People with Spina Bifida (4^th^ edition) [53] |
| Pubmed | Search 1:  Spinal cord injuries [MESH] (Limits: Guidelines/Humans/Last 10 years) | 54 | 7 | Management of pain in individuals with spinal cord injury: Guideline of the German-Speaking Medical Society for Spinal Cord Injury. [54]  Identification and Management of Cardiometabolic Risk after Spinal Cord Injury: Clinical Practice Guideline for Health Care Providers. [49]  Urodynamics in patients with spinal cord injury: A clinical review and best practice paper by a working group of The International Continence Society Urodynamics Committee. [55]  Neurogenic bowel dysfunction: Clinical management recommendations of the Neurologic Incontinence Committee of the Fifth International Consultation on Incontinence 2013. [56]  The CanPain SCI Clinical Practice Guidelines for Rehabilitation Management of Neuropathic Pain after Spinal Cord: screening and diagnosis recommendations. [57]  Professional standards of practice for psychologists, social workers, and counselors in SCI rehabilitation. [58]  Physical and rehabilitation medicine (PRM) care pathways: "spinal cord injury". [59] |
|  | Search 2: spina bifida[MESH] (Limits: Guidelines/Humans/last 10 years) | 3 | 1 | Spina Bifida Health-care Guidelines for Men's Health. [60] |
|  | **Duplicates** |  | 4 |  |
|  | **Total** |  | 34 |  |

**References**

1. Przydacz M, Chlosta P, Corcos J. Recommendations for urological follow-up of patients with neurogenic bladder secondary to spinal cord injury. Int Urol Nephrol. 2018;50:1005-1016.

2. McColl MA, Gupta S, Smith K, McColl A. Promoting long-term health among people with spinal cord injury: what’s new? International journal of environmental research and public health. 2017;14:1520.

3. Gemperli A, Ronca E, Scheel-Sailer A, Koch H, Brach M, Trezzini B. Health care utilization in persons with spinal cord injury: part 1—outpatient services. Spinal cord. 2017;55:823-827.

4. Mitchell RJ, Cameron CM, McClure R. Patterns of health care use of injured adults: a population-based matched cohort study. Injury. 2017;48:1393-1399.

5. Böthig R, Domurath B, Kaufmann A, Bremer J, Vance W, Kurze I. Neuro-urological diagnosis and therapy of lower urinary tract dysfunction in patients with spinal cord injury: S2k Guideline of the German-Speaking Medical Society of Paraplegia (DMGP), AWMF register no. 179/001. Der Urologe Ausg A. 2017;56:785-792.

6. Hossain M, Harvey L, Rahman M, Bowden J, Islam M, Taylor V, et al. A pilot randomised trial of community-based care following discharge from hospital with a recent spinal cord injury in Bangladesh. Clinical rehabilitation. 2017;31:781-789.

7. Heinemann AW, LaVela SL, Etingen B, Miskovic A, Locatelli SM, Neumann HD, et al. Perceptions of Person-Centered Care Following Spinal Cord Injury. Arch Phys Med Rehabil. 2016;97:1338-1344.

8. Van de Pol E, Lucas K, Geraghty T, Pershouse K, Harding S, Atresh S, et al. The delivery of specialist spinal cord injury services in Queensland and the potential for telehealth. BMC health services research. 2015;16:29.

9. Saur M, Abel R. Rehabilitation nach Querschnittlähmung. Der Orthopäde. 2015;44:477-488.

10. Spreyermann R, Michel F. [Long-term follow-up in patients with spinal cord injury - prevention and comprehensive care]. Praxis (Bern 1994). 2014;103:95-104.

11. Stiens SA, Fawber HL, Yuhas SA. The person with a spinal cord injury: an evolving prototype for life care planning. Phys Med Rehabil Clin N Am. 2013;24:419-444.

12. Guilcher SJ, Craven BC, Lemieux-Charles L, Casciaro T, McColl MA, Jaglal SB. Secondary health conditions and spinal cord injury: an uphill battle in the journey of care. Disability and rehabilitation. 2013;35:894-906.

13. Young‐Hughes S, Simbartl LA. Spinal cord injury/disorder teleconsultation outcome study. Rehabilitation Nursing. 2011;36:153-158.

14. Spreyermann R, Lüthi H, Michel F, Baumberger M, Wirz M, Mäder M. Long-term follow-up of patients with spinal cord injury with a new ICF-based tool. Spinal cord. 2011;49:230-235.

15. Careau E, Dussault J, Vincent C. Development of interprofessional care plans for spinal cord injury clients through videoconferencing. Journal of interprofessional care. 2010;24:115-118.

16. Morse LR, Giangregorio L, Battaglino RA, Holland R, Craven BC, Stolzmann KL, et al. VA-based survey of osteoporosis management in spinal cord injury. PM&R. 2009;1:240-244.

17. Dallolio L, Menarini M, China S, Ventura M, Stainthorpe A, Soopramanien A, et al. Functional and clinical outcomes of telemedicine in patients with spinal cord injury. Archives of Physical Medicine and Rehabilitation. 2008;89:2332-2341.

18. Bendixen RM, Levy C, Lutz BJ, Horn KR, Chronister K, Mann WC. A telerehabilitation model for victims of polytrauma. Rehabilitation Nursing. 2008;33:215-220.

19. Rintala DH, Garber SL, Friedman JD, Holmes SA. Preventing recurrent pressure ulcers in veterans with spinal cord injury: impact of a structured education and follow-up intervention. Archives of physical medicine and rehabilitation. 2008;89:1429-1441.

20. Bakketun T, Gilhus NE, Rekand T. Myelomeningocele: need for long-time complex follow-up-an observational study. Scoliosis and spinal disorders. 2019;14:3.

21. Barker RN, Sealey CJ, Polley ML, Mervin MC, Comans T. Impact of a person-centred community rehabilitation service on outcomes for individuals with a neurological condition. Disabil Rehabil. 2017;39:1136-1142.

22. Dicianno BE, Kurowski BG, Yang JM, Chancellor MB, Bejjani GK, Fairman AD, et al. Rehabilitation and medical management of the adult with spina bifida. Am J Phys Med Rehabil. 2008;87:1027-1050.

23. Khan F, Amatya B. Effectiveness of the Interdisciplinary Ambulatory Rehabilitation Program in Persons with Spina Bifida. Primary Health Care Open Access. 2015;05.

24. Szymanski KM, Misseri R, Whittam B, Large T, Cain MP. Current opinions regarding care of the mature pediatric urology patient. Journal of Pediatric Urology. 2015;11:251.e251-251.e254.

25. Veenboer PW, Procee AI, Verheijden JM, Bosch JL, van Asbeck FW, de Kort LM. Medical and psychosocial problems in middle-aged spina bifida patients: survey among members of the Dutch patients' association. Disabil Rehabil. 2014;36:539-545.

26. Böthig R, Domurath B, Bremer J, Vance W, Kaufmann A. Neuro-urologische Versorgung querschnittgelähmter Patienten. 2016.

27. Steffen F, Schulz B, Wang h, Gottschalk S, Grüter F, Friedrich J, et al. Leitlinie Schmerzen bei Querschnittlähmung. S2k.; 2018.

28. Geng V, Böthing R, Kurze I, Hildesheim A, Leder Eckhart D. S2k-Leitlinie Neurogene Darmfunktionsstörung bei Querschnittlähmung. . 2019.

29. Drzin-Schilling B, Eisenhuth J, Janker P, Neikes M, Prang P, Stolle A. S1-Leitlinie der Deutschsprachigen Medizinischen Gesellschaft für Paraplegie (DMPGP). Depression bei Menschen mit Querschnittlähmung: Besonderheiten in der Diagnostik und Behandlung. 2016.

30. Kurze I, Schmidt M, Bertschy S, Lange U, Kues S. S2k-Leitlinie 179-002. Schwangerschaft, Geburt und Wochenbett bei Frauen mit Querschnittlähmung. 2018.

31. Biglari B, Dissemond J, Donhauser M, Föcks I, Fürstenberg CH, Gstaltner K, et al. Querschnittspezifische Dekubitusbehandlung und -prävention. 2017.

32. Scheel-Sailer A, Bartholet C, Bersch-Porada I, Curt A, Gisler A, Huber B, et al. Rehabilitation der unteren Exremität, der Steh- und Gehfunktion bei Menschen mit Querschnittlähmung. 2018.

33. Kalke Y-B, Frotzer A, Moosburger J, Wittgruber G. Querschnittlähmungsassoziierte Osteoporose. 2018.

34. Stein R, Assion C, Bredel Geissler A, Beetz R, Bürst M, Cremer R, et al. Diagnostik und Therapie der neurogenen Blasenfunktionsstörungen bei Kindern und Jugendlichen mit spinaler Dysraphie. S2k Leitlinie 043-047. . 2013.

35. SCIRE Professional. Rehabilitation Evidence 2010 [Available from: <https://scireproject.com/evidence/rehabilitation-evidence/>.

36. NICE, National Institute for Health and Care Excellence. Urinary incontinence in neurological disease: assessment and management. 2012.

37. NICE, National Institute for Health and Care Excellence. Faecal incontinence in adults: management. 2007.

38. NICE, National Institute for Health and Care Excellence. Pressure ulcers: prevention and management. United Kingdom; 2014.

39. British Society of Rehabilitation Medicine. BSRM Standards for Rehabilitation Services mapped on to the National Service Framework for Long-term neurological conditions. 2009.

40. Consortium for Spinal Cord Medicine, Paralyzed Veterans of America. Prevention of Venous Thromboembolism in Individuals with Spinal Cord Injury: Clinical Practice Guideline for Health Care Providers. 2016.

41. Consortium for Spinal Cord Medicine, Paralyzed Veterans of America. Sexuality and Reproductive Health in Adults with Spinal Cord Injury: A Clinical Practice Guideline for Health-Care Providers. 2010.

42. Consortium for Spinal Cord Medicine, Paralyzed Veterans of America. Bladder Management for Adults with Spinal Cord Injury: A Clinical Practice Guideline for Health-Care Providers. 2006.

43. Consortium for Spinal Cord Medicine, Paralyzed Veterans of America. Preservation of upper limb function following spinal cord injury: a clinical practice guideline for health-care professionals. The journal of spinal cord medicine. 2005;28:434-470.

44. Consortium for Spinal Cord Medicine, Paralyzed Veterans of America. Respiratory Management Following Spinal Cord Injury: A Clinical Practice Guideline for Health-Care Providers. 2005.

45. Consortium for Spinal Cord Medicine, Paralyzed Veterans of America. Pressure Ulcer Prevention and Treatment Following Spinal Cord Injury: A Clinial Pracitce Guideline for Health-Care Professionals. Washington, DC: Paralyzed Veterans of America; 2014.

46. Consortium Guidelines for Spinal Cord Medicine, Paralyzed Veterans of America. Outcomes Following Traumatic Spinal Cord Injury: Clinical Practice Guidelines for Health-Care Professionals. 1999.

47. Consortium for Spinal Cord Medicine, Paralyzed Veterans of America. Depression Following Spinal Cord Injury: A Clinical Practice Guideline for Primary Care Physicians: Consortium for Spinal Cord Medicine; 1998.

48. Consortium for Spinal Cord Medicine, America PVo. Clinical practice guidelines: Neurogenic bowel management in adults with spinal cord injury. Spinal Cord Medicine Consortium. J Spinal Cord Med. 1998;21:248-293.

49. Nash MS, Groah SL, Gater DR, Jr., Dyson-Hudson TA, Lieberman JA, Myers J, et al. Identification and Management of Cardiometabolic Risk after Spinal Cord Injury: Clinical Practice Guideline for Health Care Providers. Top Spinal Cord Inj Rehabil. 2018;24:379-423.

50. Coggrave M, Norton C, Cody JD. Management of faecal incontinence and constipation in adults with central neurological diseases. Cochrane Database of Systematic Reviews. 2014.

51. Joyce P, Moore ZE, Christie J. Organisation of health services for preventing and treating pressure ulcers. Cochrane Database Syst Rev. 2018;12:Cd012132.

52. Posadzki P, Mastellos N, Ryan R, Gunn LH, Felix LM, Pappas Y, et al. Automated telephone communication systems for preventive healthcare and management of long-term conditions. Cochrane Database Syst Rev. 2016;12:Cd009921.

53. Spina Bifida Association. Guidelines for the Care of People with Spina Bifida 2018 [Available from: <https://www.spinabifidaassociation.org/guidelines/>.

54. Steffen F, Schulz B, Wang H, Gottschalk S, Grüter F, Friedrich J, et al. Management of pain in individuals with spinal cord injury: Guideline of the German-Speaking Medical Society for Spinal Cord Injury. Ger Med Sci. 2019;17:Doc05.

55. Schurch B, Iacovelli V, Averbeck MA, Stefano C, Altaweel W, Finazzi Agrò E. Urodynamics in patients with spinal cord injury: A clinical review and best practice paper by a working group of The International Continence Society Urodynamics Committee. Neurourol Urodyn. 2018;37:581-591.

56. Cotterill N, Madersbacher H, Wyndaele JJ, Apostolidis A, Drake MJ, Gajewski J, et al. Neurogenic bowel dysfunction: Clinical management recommendations of the Neurologic Incontinence Committee of the Fifth International Consultation on Incontinence 2013. Neurourol Urodyn. 2018;37:46-53.

57. Mehta S, Guy SD, Bryce TN, Craven BC, Finnerup NB, Hitzig SL, et al. The CanPain SCI Clinical Practice Guidelines for Rehabilitation Management of Neuropathic Pain after Spinal Cord: screening and diagnosis recommendations. Spinal cord. 2016;54 Suppl 1:S7-s13.

58. Russell HF, Richardson EJ, Bombardier CH, Dixon TM, Huston TA, Rose J, et al. Professional standards of practice for psychologists, social workers, and counselors in SCI rehabilitation. J Spinal Cord Med. 2016;39:127-145.

59. Albert T, Beuret Blanquart F, Le Chapelain L, Fattal C, Goossens D, Rome J, et al. Physical and rehabilitation medicine (PRM) care pathways: "spinal cord injury". Ann Phys Rehabil Med. 2012;55:440-450.

60. Wiener JS, Frimberger DC, Wood H. Spina Bifida Health-care Guidelines for Men's Health. Urology. 2018;116:218-226.
